# Duration Adaptation Occurs Across the Sub- and Supra-Second Systems

**DOI:** 10.3389/fpsyg.2016.00114

**Published:** 2016-02-09

**Authors:** Shuhei Shima, Yuki Murai, Yuki Hashimoto, Yuko Yotsumoto

**Affiliations:** ^1^Department of Integrated Sciences, The University of TokyoTokyo, Japan; ^2^Department of Life Sciences, The University of TokyoTokyo, Japan

**Keywords:** time perception, duration, sub-second, supra-second, time reproduction, adaptation

## Abstract

After repetitive exposure to a stimulus of relatively short duration, a subsequent stimulus of long duration is perceived as being even longer, and after repetitive exposure to a stimulus of relatively long duration, a subsequent stimulus of short duration is perceived as being even shorter. This phenomenon is called duration adaptation, and has been reported only for sub-second durations. We examined whether duration adaptation also occurs for supra-second durations (Experiment 1) and whether duration adaptation occurs across sub- and supra-second durations (Experiment 2). Duration adaptation occurred not only for sub-second durations, but also for supra-second durations and across sub- and supra-second durations. These results suggest that duration adaptation involves an interval-independent system or two functionally related systems that are associated with both the sub- and supra-second durations.

## Introduction

Perceived duration of an event sometimes deviates from its physical duration. This so-called ‘time distortion’ (Herbst et al., 2013; Kliegl and Huckauf, 2014) has been a useful tool for understanding time-processing mechanisms. The phenomenon of duration adaptation means that after repetitive exposure to a stimulus of relatively short duration, a subsequent stimulus of long duration is perceived as being even longer, and after repetitive exposure to a stimulus of relatively long duration, a subsequent stimulus of short duration is perceived as being even shorter (Walker et al., 1981; Heron et al., 2012). Generally, adaptation is an ecologically useful mechanism, which enables us to flexibly and rapidly change our sensitivity to external stimuli according to the environment around us. Adaptation methods have been widely applied in studies of sensory perception to investigate the mechanisms underlying dynamic and environment-dependent adjustments to our perceptual processes (Webster, 2011). Adaptation-induced time distortion is analogous to sensory adaptation, and is a powerful tool for investigating time-processing mechanisms.

Indeed, many studies have investigated the time-processing mechanisms associated with this duration adaptation paradigm. Heron et al. (2012) proposed the existence of duration-selective channels that respond selectively to a narrow range of stimulus durations centered on a preferred duration. It was suggested that these channels cause duration adaptation. Walker et al. (1981) revealed that duration adaptation does not transfer across modalities, and Heron et al. (2013) reported that duration adaptation precedes multisensory integration. These results implicate modality-dependent clock systems, and suggest that duration adaptation occurs at relatively early stages in sensory processing. Conversely, it has been shown that visual duration adaptation is contingent neither on the orientation of the stimulus (Li et al., 2015a) nor on the hemifield of stimulus presentation (Li et al., 2015b). These results alternatively suggest that duration adaptation occurs at a relatively later stage in sensory processing. Previous studies have reported that duration adaptation occurs only for durations less than 1 s (i.e., sub-second). As aforementioned, most studies suggest that duration adaptation is related to sensory processing, and assume that duration adaptation is a phenomenon limited to the sub-second range, which is crucial for sensory perception. However, duration adaptation for durations of greater than 1 s (i.e., supra-second) has not been explicitly tested in previous studies, and therefore it remains unclear whether duration adaptation occurs for the supra-second time range.

Several studies have suggested that different time-processing systems are used depending on the stimulus duration. For example, it is widely accepted that there are two distinct time-processing systems divided at a boundary of about 1 s (Buhusi and Meck, 2005; Gooch et al., 2011). The involvement of different neural networks for sub- and supra-second processing was reported by studies that used different methods such as fMRI (Lewis and Miall, 2003a,b; Wiener et al., 2010), neuroanatomy (Hayashi et al., 2014), transcranial magnetic stimulation (TMS; Koch et al., 2007), and neuropsychology (Gooch et al., 2011). A pharmacological study showed that drugs that impair the processing of sub-second durations do not impair the processing of supra-second durations, and conversely, drugs that impair the processing of supra-second durations do not impair the processing of sub-second durations (Rammsayer, 1999). Furthermore, unique genotypes are associated with sub-second versus supra-second duration processing (Wiener et al., 2011). Psychophysical analyses have revealed that the individual Weber fraction for sub-second durations does not correlate with that of supra-second durations (Hayashi et al., 2014). Additionally, increases in cognitive load selectively affect performance in a temporal task of supra-second duration (Rammsayer and Lima, 1991). These differences between the sub-second and supra-second processing systems suggest that duration adaptation in sub-second durations may not apply to duration adaptation in supra-second durations.

A system that functions across the sub- and supra-second durations, termed the *interval-independent system*, has also been proposed (Rammsayer and Troche, 2014). The traditional pacemaker-accumulator model assumes that there exists a single timing mechanism (Treisman, 1963). Lewis and Miall (2009) showed that precision (the coefficient of variation of the perceived duration) decreases constantly across the sub- and supra-second durations without any “break point”. Such continuous perceptual characteristics implicate an interval-independent timing mechanism. Another study reported that central tendency also occurs across the sub-second and supra-second durations (Lejeune and Wearden, 2009). Central tendency describes a phenomenon in which relatively short durations are likely to be overestimated and relatively long durations are likely to be underestimated in tests of multiple durations. The observation of central tendency across the sub- and supra-second durations suggests that multiple durations were processed in an interval-independent system. If systems that function across the sub- and supra-second durations are responsible for duration adaptation, then it follows that duration adaptation should also be observed for supra-second durations.

In the present study, we investigated how duration adaptation affects the perceived duration of the sub- and the supra- second stimuli, and whether these effects remain within the sub- or the supra-second systems, or extend across the systems. We propose three possible patterns for duration adaptation. First, duration adaptation may occur only in the sub-second durations and not in the supra-second durations. This scenario would suggest that duration adaptation involves only the sub-second system. Second, duration adaptation may occur in the sub-second durations and in the supra-second durations, but may not occur across both the sub- and supra-second durations. If this is the case, the supra-second duration would not be perceived as being longer after adapting to the sub-second duration, and the sub-second duration would not be perceived as being shorter after adapting to the supra-second duration. This scenario would indicate that duration adaptation affects the two distinct systems independently. Third, duration adaptation occurs in the sub-second durations, in the supra-second durations, and across the sub- and supra-second durations. This scenario would indicate that duration adaptation involves systems extending across the sub- and supra-second systems.

In Experiment 1, we used sub-second durations for the adapting and test stimuli, aiming to replicate previous studies. We also used supra-second durations to examine whether duration adaptation occurs within supra-second durations. In Experiment 2, we used sub- and supra-second durations to examine whether duration adaptation occurs across the sub- and supra-second durations.

## Experiment 1 Duration Adaptation Within Sub- And Supra-Second Durations

The aim of this experiment was to examine whether duration adaptation occurs in sub-second durations and in supra- second durations.

### Materials and Methods

#### Participants

Seven students of the University of Tokyo (two authors and five naïve participants, two female, average age 20.8 ± 1.2 years) participated in this experiment. All participants gave written informed consent for their participation in the experimental protocol, which was approved by the institutional review boards of The University of Tokyo. The participants were all right handed and with normal or corrected-to-normal vision.

#### Apparatus

Stimuli were generated using Matlab and the Psychophysics-toolbox (Brainard, 1997; Pelli, 1997) and presented on a gamma-corrected CRT monitor (DiamondtronM2 RDF223H, Mitsubishi) controlled by iMac OS X 10.9.5 (Apple, 1024 × 768 pixels, 120 Hz refresh rate). The experiment was conducted in a dark room. The viewing distance was 57.3 cm and participants were asked to stabilize their head on a chin rest.

#### Procedure

**FIGURE 1:**
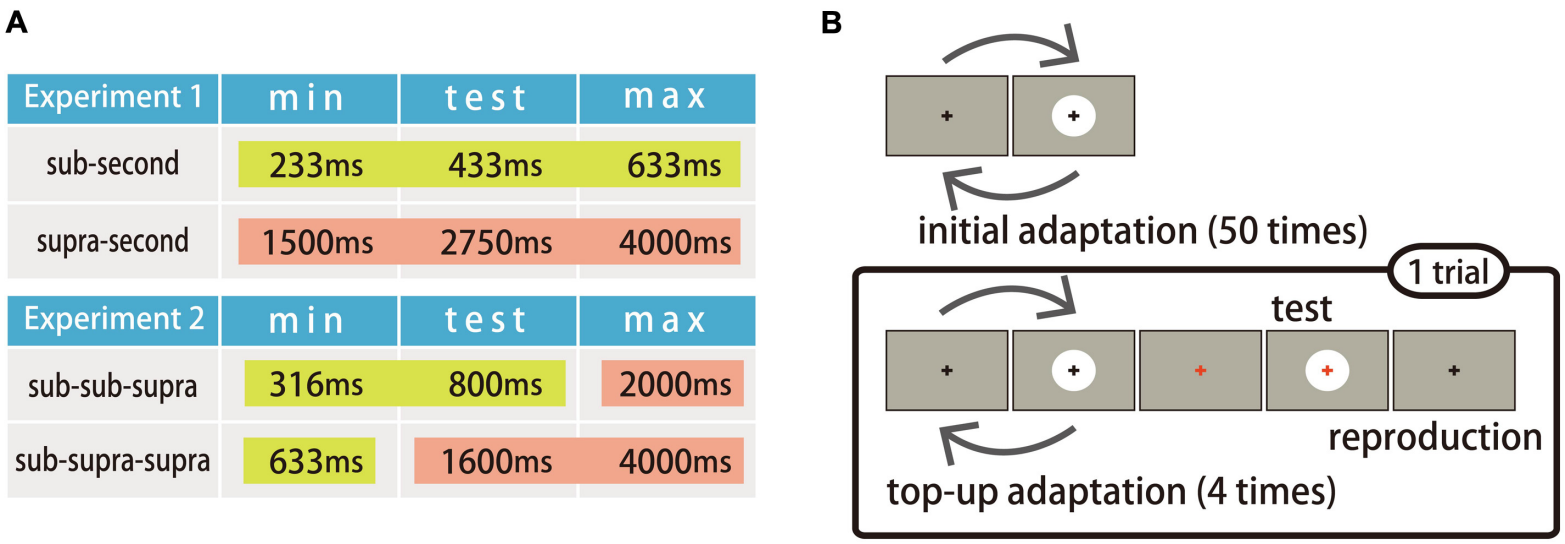
**Experimental design. (A)** The minimum adapting durations, the test durations, and the maximum adapting durations used in each condition and experiment. Yellow boxes represent sub-second durations and red boxes represent supra-second durations. **(B)** Experimental procedures used in Experiments 1 and 2.

Two conditions, the sub-second and the supra-second, were tested in Experiment 1. In each condition, the duration of the test stimulus (test duration) was fixed and the durations of the adapting stimuli (adapting durations) were varied in seven steps. Among the seven adapting durations, three were shorter than the test duration, three were longer than the test duration, and one was the same length as the test duration. **Figure 1A** illustrates the minimum adapting duration, test duration, and the maximum adapting duration used in each condition (see Stimuli). In the sub-second condition, the minimum adapting duration, test duration, and maximum adapting duration were all in the sub-second range. In the supra-second condition, on the other hand, the minimum adapting duration, test duration, and maximum adapting duration were all in the supra-second range. The sub-second condition was conducted to replicate the duration adaptation reported in previous studies (Walker et al., 1981; Heron et al., 2012).

The sub-second condition was tested in one session, and the supra-second condition was tested in four sessions. The order of the sub-second condition and the supra-second condition was counterbalanced across participants. Each session consisted of seven blocks, in each of which the adapting duration remained constant. Each block consisted of an initial adaptation and test trials. The number of test trials in one block was sixty in the sub-second condition, and fifteen in the supra-second condition, resulting in each adapting duration being tested sixty times for each condition. The order of blocks within each session was randomized within and between the participants.

**Figure 1B** illustrates the experimental procedure. At the beginning of each block, the participants first observed the adapting stimulus fifty times as an initial adaptation. The test trials followed the initial adaptation. One trial consisted of four top-up adaptations, a test stimulus presentation, and a reproduction of the test duration. The inter-stimulus interval (ISI) between the initial adaptation and the first trial was 2000 ms, and other ISIs (between the adapting stimuli and between the adapting and the test stimuli) were randomly jittered in the range of 500–1000 ms.

Participants were asked to reproduce the test duration by pressing a button with the forefinger of their dominant hand. They were instructed to make responses as accurately as possible after the color of the fixation point changed from black to red. They were also instructed to fixate on the point at the center of the stimuli, and not to count the durations (Rattat and Droit-Volet, 2012).

Before each session, the participants underwent a practice block to accustom themselves to the reproduction task procedure. The practice block contained neither an initial adaptation nor top–up adaptations, and consisted of 60 trials for the sub-second condition and 15 trials for the supra-second condition.

#### Stimuli

A white disk (88 cd/m^2^, 5° in diameter) on a gray background (18 cd/m^2^, 40 × 30°) was used as both an adapting and test stimulus. The stimuli were presented at the center of the screen. The fixation point was a black or a red cross (1 × 1°) presented at the center of the screen throughout the sessions. In the sub-second condition, the test duration was 433 ms and the adapting duration was 233, 316, 400, 433, 466, 550, or 633 ms. In the supra-second condition, the test duration was 2750 ms and the adapting duration was 1500, 2000, 2500, 2750, 3000, 3500, or 4000 ms.

### Results and Discussion

The reproduced durations were regressed to the logarithm of adapting duration (Heron et al., 2012) with a first degree polynomial equation (Walker et al., 1981).

$$reproduced\,\;duration=\beta_{0}\times \log_{10}(adapting\,\;duration)+\beta_{1}$$

β_0_ and β_1_ were calculated for each participant. To test whether the across-participants-mean of the slope coefficients *β_0_* (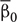) was significantly negative, bootstrap resampling was conducted. Bootstrap samples of 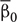 were calculated through resampling of participants and their reproduced duration with replacement over 10000 iteration. For the null-hypothesis that the true value of 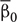 was not negative, a *p*-value was calculated as the proportion of iteration in which the resampled 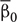 was not negative.

Trials in which the reaction time was beyond the median ± 3 *SD*s (Miller, 1991), trials in which a reaction time was less than 100 ms (reaction too early), and trials in which the reproduced duration was beyond the median ± 3 *SD*s were removed from the analysis. This resulted in 2.2% of the total trials in the sub-second condition and 1.7% of the total trials in the supra-second condition being excluded from further analyses.

**FIGURE 2:**
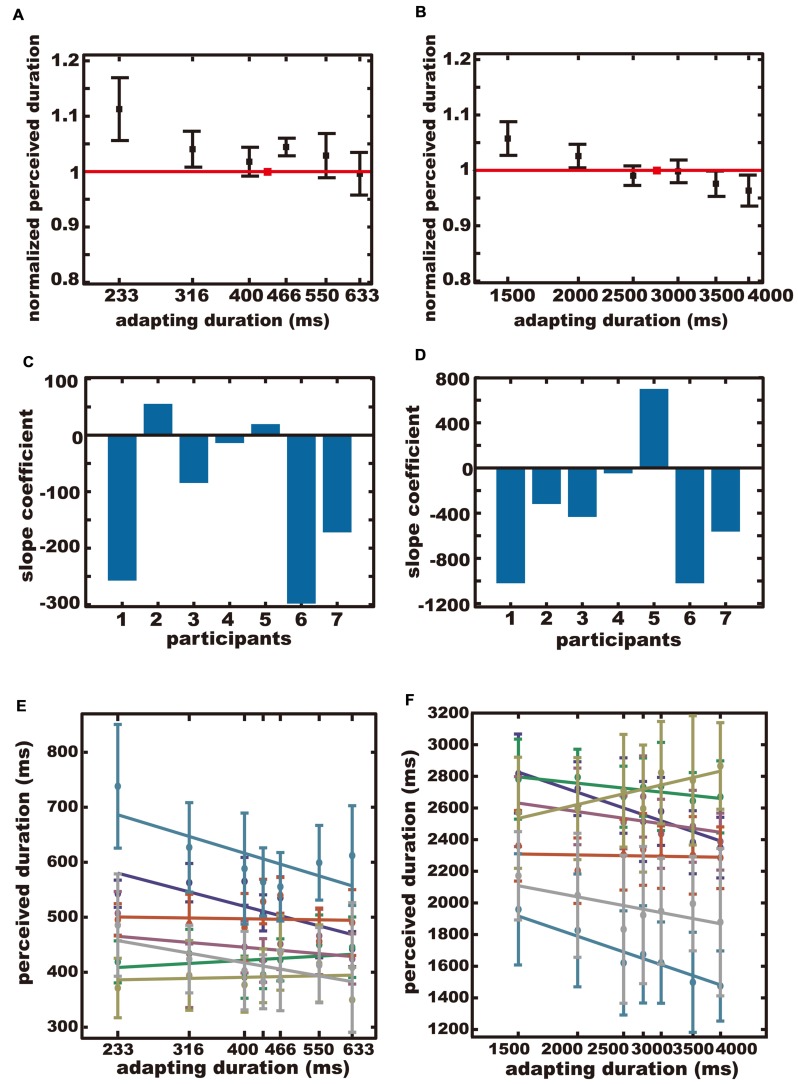
**The results of Experiment 1. (A,B)** The average normalized perceived durations. The red square indicates the normalized perceived duration after adapting to the same duration as the test duration. Error bars indicate standard errors (*SE*). **(C,D)** Regression coefficients for all participants. **(E,F)** Perceived durations plotted separately for each participant. Each color represents each participant. **(A,C,E)** The sub-second condition. **(B,D,F)** The supra-second condition.

If the adapting duration and the test duration were the same, there would be no aftereffect. Hence, for each participant, we computed the mean of the reproduced duration for this particular adapting duration, and divided the reproduced duration for the other adapting durations by that particular reproduced duration. We refer to this normalized reproduced duration as the *normalized perceived duration*. **Figure 2** shows the across-subjects average of the normalized perceived duration.

If duration adaptation occurs, the normalized perceived duration would be larger after adapting to shorter durations, and would be smaller after adapting to longer durations, resulting in a negative trend for the adapting duration. If duration adaptation does not occur, the normalized perceived duration would be constant regardless of the adapting duration.

The average normalized perceived durations were plotted separately for the sub-second condition (**Figure 2A**), and for the supra-second condition (**Figure 2B**). A negative trend was observed for both conditions. The across-participant mean of the reproduced duration when the test and adapting durations were the same, was 456 ± 69 ms (*SD*) in the sub-second condition, and 2350 ± 373 ms (*SD*) in the supra-second condition.

The slope coefficient for each participant is shown in **Figures 2C,D** for the sub-second condition and the supra-second condition, respectively. The perceived duration for each subject for each adapting duration is shown in **Figures 2E,F** for the sub-second condition and the supra-second condition, respectively. The normalized perceived duration for each subject for each adapting duration is shown in Figures S1A,B, for the sub-second condition and the supra-second condition, respectively. The means of the slope coefficients were –107 in the sub-second condition and –386 in the supra-second condition. Bootstrap testing confirmed that the across-participants-mean of the slope coefficients were significantly negative in the sub-second condition (*p* = 0.01), and in the supra-second condition (*p* = 0.04). In other words, a significant aftereffect was observed both in the sub-second and the supra-second ranges.

In this experiment, unlike some previous studies (Walker et al., 1981; Heron et al., 2012, 2013; Li et al., 2015a,b), several participants in the sub-second condition did not exhibit an aftereffect after adapting to 466, 550, and 633 ms. One possible explanation could be related to the task we used. In our experiment, the participants reproduced the test duration, whereas a previous study (Heron et al., 2012) used a discrimination task. Due to the sustained button press required during the response, the reproduction task might be sensitive to motor noise that could contribute to the large variance for short durations (Wearden, 2003), and this could have weakened the aftereffect. It has also been reported that participants in a reproduction task are biased to reproduce a duration that is longer than the actual duration, and the bias is more prominent with shorter durations (Shi et al., 2013). The time difference between the test duration and the maximum adapting duration could provide another plausible explanation. The range of the adapting durations tested in the present study was smaller than that used in the previous study (Heron et al., 2012). A larger range of adapting durations could have induced a larger aftereffect. Nonetheless, the normalized perceived durations exhibited significant negative trends for both sub- and supra- second durations, indicating significant aftereffects for both time ranges.

In Experiment 1, duration adaptation occurred not only for sub-second durations but also for supra-second durations. In Experiment 2, we examined whether the duration adaptation occurs across sub- and supra-second durations.

## Experiment 2 Duration Adaptation Across Sub- And Supra-Second Durations

The aim of this experiment was to examine whether duration adaptation occurs across the sub- and supra- second durations.

### Materials and Methods

#### Participants

Five students of the University of Tokyo (one author and four naïve participants, two left handed, average age 20.6 ± 0.8 years) participated in this experiment. All participants gave written informed consent for their participation in the experimental protocol, which was approved by the institutional review boards of The University of Tokyo. The participants were all males and with normal or corrected-to-normal vision.

#### Apparatus

The same apparatus was used as in Experiment 1.

#### Procedure

Two conditions were tested in Experiment 2: the sub-sub-supra, and the sub-supra-supra. In each condition, the test duration was fixed and the adapting durations were varied in five steps. Among the five adapting durations, two were shorter than the test duration, two were longer than the test duration, and one was same as the test duration. **Figure 1A** illustrates the minimum adapting duration, test duration, and maximum adapting duration for each condition. In the condition named ‘sub-sub-supra’, the minimum adapting duration and the test duration were in the sub-second range, and the maximum adapting duration was in the supra-second range. This condition was designed to examine whether duration adaptation occurs for sub-second durations after adapting to supra-second durations. In the other condition named ‘sub-supra-supra’, the minimum adapting duration was in the sub-second range, while the test duration and the maximum adapting duration were in the supra-second range. This condition was designed to examine whether the duration adaptation occurs for supra-second durations after adapting to sub-second durations.

The sub-sub-supra condition was tested in one session, and the sub-supra-supra condition was tested in two sessions. The order of the sub-sub-supra and sub-supra-supra conditions was counterbalanced across participants. Each session consisted of five blocks, in each of which the adapting duration remained constant. Each block consisted of an initial adaptation and test trials. The number of test trials in one block was 60 for the sub-sub-supra condition, or thirty for the sub-supra-supra condition, resulting in each adapting duration being tested 60 times for each condition.

The experimental procedure was the same as in Experiment 1 (**Figure 1B**). The order of blocks within each session was counterbalanced within and between participants using a Latin square design.

Before each session, the participants underwent a practice block. The practice block contained neither an initial adaptation nor top–up adaptations, and consisted of sixty trials for the sub-sub-supra condition and thirty trials for the sub-supra-supra condition.

#### Stimuli

The same stimuli were used as in Experiment 1 apart from the adapting and test durations. In the sub-sub-supra condition, the test duration was 800 ms and the adapting durations were 316, 500, 800, 1250, and 2000 ms. In the sub-supra-supra condition, the test duration was 1600 ms and the adapting durations were 633, 1000, 1600, 2500, and 4000 ms.

### Results and Discussion

The reproduced durations were regressed to the logarithm of adapting duration (Heron et al., 2012) with a first degree polynomial equation (Walker et al., 1981; see Results and Discussion). β_0_ and β_1_ were calculated for each participant. Bootstrap testing was conducted to determine whether 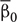 was significantly negative.

Several trials were excluded from the analysis using the same criteria as in Experiment 1, resulting in 2.7% of the total trials in the sub-sub-supra condition and 2.3% of the total trials in the sub-supra-supra condition being excluded from further analyses.

**FIGURE 3:**
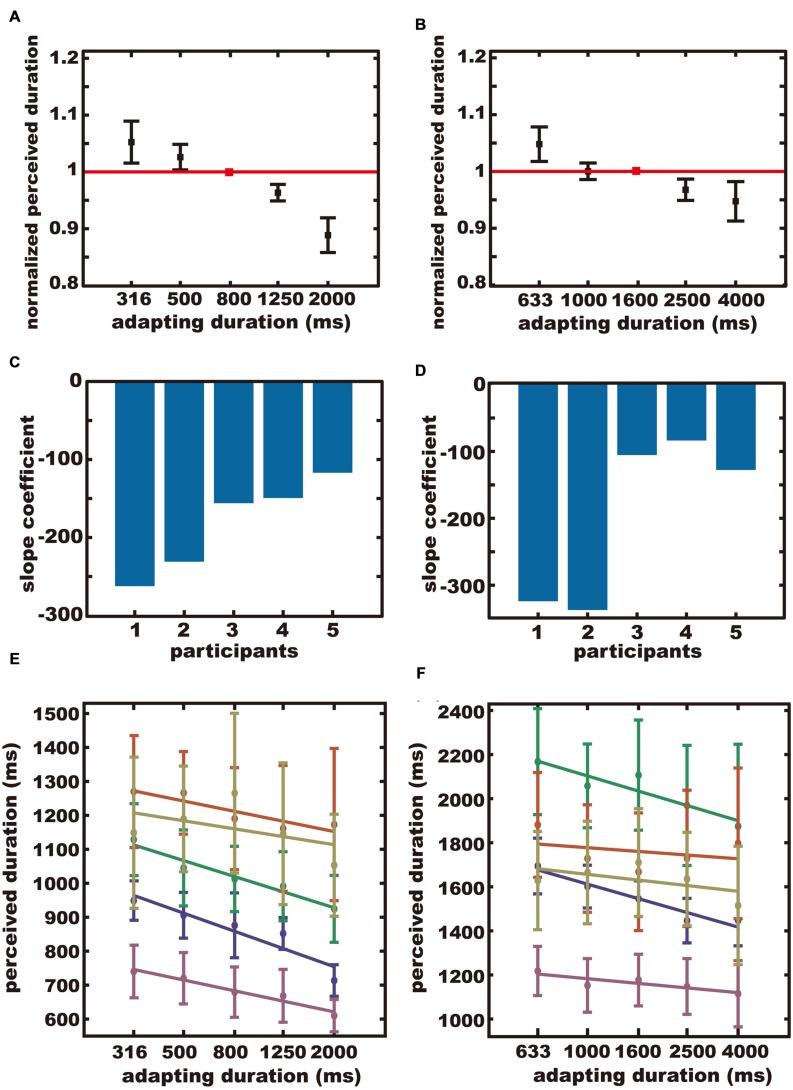
**The results of Experiment 2. (A,B)** The averaged normalized perceived durations. The red square indicates the normalized perceived duration after adapting to the test duration. Error bars indicate *SE*. **(C,D)** Regression coefficients for all participants. **(E,F)** Perceived durations plotted separately for each participant. Each color represents each participant. **(A,C,E)** The sub-sub-supra condition. **(B,D,F)** The sub-supra-supra condition.

The averaged normalized perceived durations were plotted separately for the sub-sub-supra condition (**Figure 3A**), and the sub-supra-supra condition (**Figure 3B**). In both conditions, the test duration was perceived as being longer after adapting to a short duration and shorter after adapting to a long duration. The across-participant mean of the reproduced duration when the test and adapting durations were the same, was 1005 ± 212 ms (*SD*) in the sub-sub-supra condition, and 1641 ± 300 ms (*SD*) in the sub-supra-supra condition.

The slope coefficient for each participant is shown in **Figures 3C,D** for the sub-sub-supra condition and the sub-supra-supra condition, respectively. The perceived duration for each subject for each adapting duration is shown in **Figures 3E,F** for the sub-sub-supra condition and the sub-supra-supra-second condition, respectively. The normalized perceived duration for each subject for each adapting duration is shown in Figures S1C,D, for the sub-sub-supra condition and the sub-supra-supra-second condition, respectively. The means of the slope coefficients were –183 in the sub-sub-supra condition and –195 in the sub-supra-supra condition. Bootstrap testing confirmed that the across-participants-mean of the slope coefficients were significantly negative in the sub-sub-supra condition (*p* < 0.001), and in the sub-supra-supra condition (*p* < 0.001). In other words, a significant aftereffect was observed for the sub-second duration after adapting to supra-second durations, and for the supra-second duration after adapting to sub-second durations.

## General Discussion

Our results indicate that duration adaptation occurred not only for sub-second durations but also for supra-second durations, and occurred across the sub- and supra-second durations.

Previous studies have reported duration adaptation only for sub-second durations (Walker et al., 1981; Heron et al., 2012). Their results imply that duration adaptation only involves the sub-second system. However, our results from Experiment 1 disproved that possibility. Our results from Experiment 2 also denied the possibility that duration adaptation affects the two distinct systems independently. Our results suggest that duration adaptation involves both the sub- and supra-second systems.

Rammsayer and Troche (2014) proposed a model of interval timing using a statistical approach and suggested two possibilities for time-processing systems. Based on their theory, we propose two possible alternatives to explain how duration adaptation involves time processing systems. One explanation is that duration adaptation involves an interval-independent system, not two distinct systems. This is the simplest explanation that is in accord with our results. This explanation is based on the possibility proposed by Rammsayer and Troche (2014) that temporal information is processed by two distinct systems at the initial level, while both these systems are controlled by a common, interval-independent superordinate processing system at the next level. Our alternative explanation is that duration adaptation involves both the sub- and supra-second systems, which are not distinct but are associated with each other. This explanation is based on the possibility proposed by Rammsayer and Troche (2014) that the sub- and supra-second systems are functionally related. That is, durations near 1 s are not processed by either the sub- or supra-second systems, but are processed by both the sub- and supra-second systems, and the boundary between the sub- and the supra-second system is not fixed at 1 s, but is more continuous. It should be noted that these two explanations are not mutually exclusive. In either case, the idea of common or continuous mechanisms between sub- and supra-second systems are also supported by fMRI studies indicating that various brain regions including the inferior frontal gyrus, the somatosensory area, and the supplementary motor area process both sub- and supra-second durations (Lewis and Miall, 2003a; Morillon et al., 2009; Wiener et al., 2010).

Based on previous studies (Ivry, 1996; Becker and Rasmussen, 2007), Heron et al. (2012) suggested that there are duration channels that respond selectively to a narrow range of stimulus durations centered on their preferred duration, and that changes in the channels’ responses cause the aftereffect. This model is called the channel-based model (Heron et al., 2012). If this model explains duration adaptation, the largest aftereffect would be observed when the difference between the adapting and the test duration is optimal, and the aftereffect magnitude would become smaller when the difference becomes larger or smaller than the optimal value. This type of aftereffect is called ‘repulsion-type aftereffect’ (Schwartz et al., 2007), and was not observed in our experiments. However, it should be noted that our results do not refute the channel-based model. In the present study, the difference between the adapting and the test durations is smaller than in Heron’s study. The ratio of test duration to minimum adapting duration and the ratio of maximum adapting duration to test duration were eight in Heron’s study, while the ratios were 2 or 1.4 in Experiment 1, and 2.5 in Experiment 2 of our study. This difference could explain why the ‘repulsion-type aftereffect’ was not observed in the present study.

The parameters used in our study were selected based on the assumption that the boundary between the sub- and supra-second systems is around 1 s (Buhusi and Meck, 2005; Gooch et al., 2011). Some researchers have reported the boundary at around 500 ms (Rammsayer and Lima, 1991; Rammsayer, 1999), while others have reported the boundary at around 2 s (Morillon et al., 2009). Even if the boundary is not around 1 s, our conclusion that duration adaptation occurs across the sub- and supra-second systems is still justified. If the boundary is 500 ms, we can say that, from the sub-sub-supra condition in Experiment 2, an aftereffect was observed for the supra-second duration after adapting to sub-second durations. Conversely, we cannot say that an aftereffect was observed for the sub-second duration after adapting to supra-second durations. However, previous studies have reported that such an aftereffect occurs. For example, Heron et al. (2012) showed that duration adaptation occurs for 320 ms after adapting to 640 ms. Therefore, we can speculate that an after effect would be observed for the sub-second duration after adapting to supra-second durations. Alternatively, if the boundary is 2 s, we can say that an aftereffect was observed for the sub-second duration after adapting to supra-second durations, from the sub-supra-supra condition in Experiment 2. We can also say that, from the supra-second condition in Experiment 1, the aftereffect was also observed for the supra-second duration after adapting to sub-second durations.

Previous studies have suggested that duration adaptation is associated with relatively early stages in sensory processing because duration adaptation precedes multisensory integration (Heron et al., 2013) and does not transfer across modalities (Walker et al., 1981). Additionally, sub-second duration tuning cells are found in primary sensory areas such as the primary visual cortex in the cat (Duysens et al., 1996) and in subcortical structures such as the inferior colliculus in the bat (Casseday et al., 1994; Faure et al., 2003). Here, we propose that duration adaptation is also associated with stages that involve both sub- and supra-second durations. The present study indicates that duration adaptation spans a millisecond-to-second time scale. Duration-tuned cells for the supra-second range are found in the prefrontal cortex in primates (Yumoto et al., 2011). If duration-selective cells act as duration channels and cause adaptation, it follows that duration adaptation should occur in the supra-second range. A recent fMRI study showed that the inferior parietal lobule exhibits duration-selective neural activity (Hayashi et al., 2015). These studies imply that the neural induction of duration adaptation is not restricted to subcortical structures or primary sensory areas that play key roles in primary sensory processing, but is rather distributed among the various brain regions that comprise timing networks. These multiple brain areas may work cooperatively as a network and consist of a single interval-independent mechanism. The present study provides psychophysical evidence for a general duration-selective mechanism in the human brain.

## Author Contributions

Conceived the experiments: SS, YM, YH, YY. Designed the experiments: SS, YM. Performed the experiments: SS. Analyzed the data: SS, YH. Wrote the paper: SS, YM, YH, YY.

## Conflict of Interest Statement

The authors declare that the research was conducted in the absence of any commercial or financial relationships that could be construed as a potential conflict of interest.
