# Supplementary material for: Duration Adaptation Occurs Across the Sub- and Supra-Second Systems
Source: Front Psychol. 2016 Feb 9;7:114. doi: 10.3389/fpsyg.2016.00114 (PMC4746325; doi:10.3389/fpsyg.2016.00114)
Supplement: Supplementary file 1 [file Data_Sheet_1.DOCX]

Supplementary Material

**Duration adaptation occurs across the sub- and supra-second systems**

**Shuhei Shima 1, Yuki Murai 2, Yuki Hashimoto 2, and Yuko Yotsumoto 2***

*** Correspondence:** Yuko Yotsumoto, cyuko@mail.ecc.u-tokyo.ac.jp

**1. Supplementary Figure**


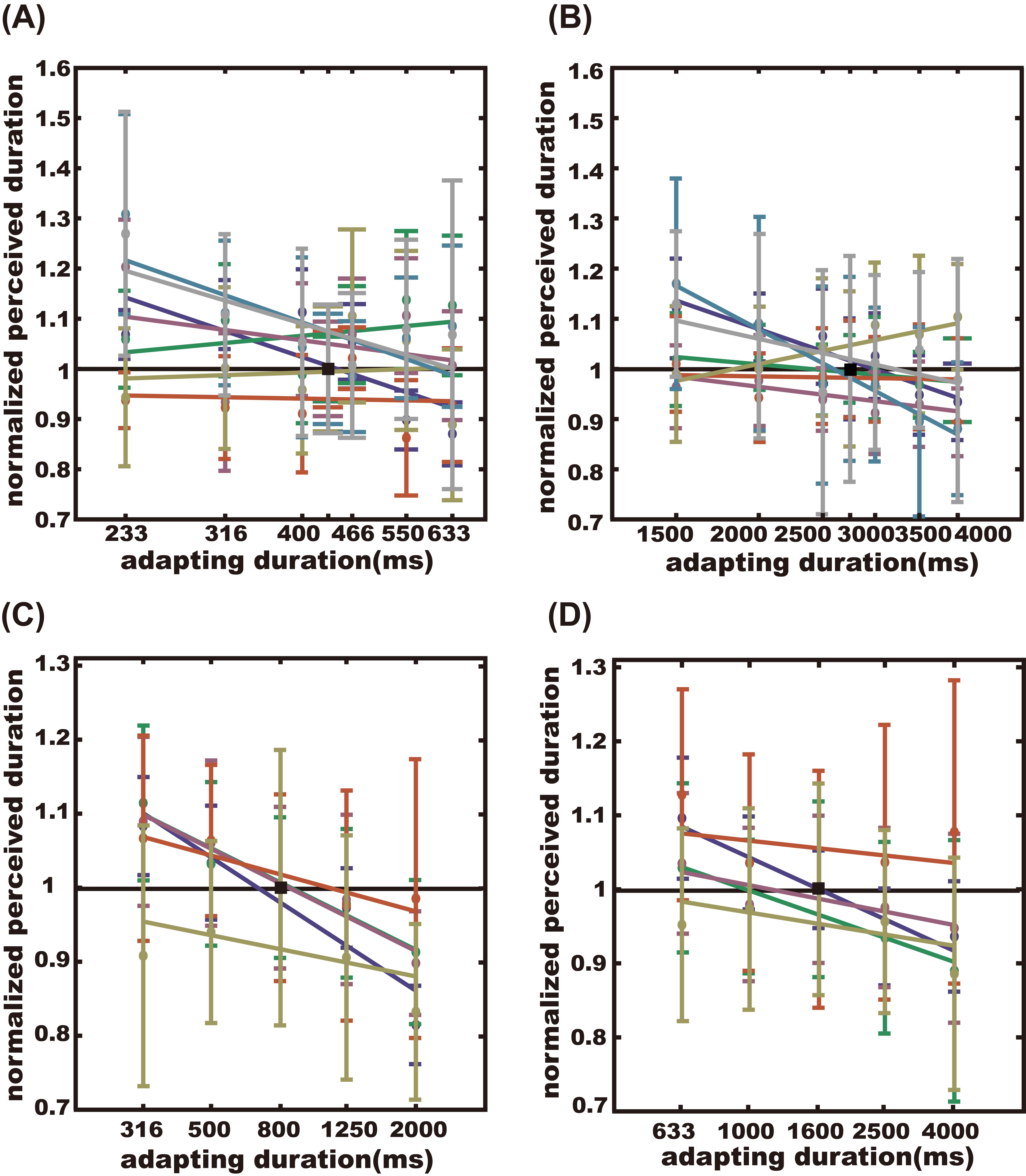


Supplementary Figure 1. Normalized perceived durations plotted separately for each participant. Each color represents each participant. Error bars indicate SE. (A) The sub-second condition in Experiment 1. (B) The supra-second condition in Experiment 1. (C) The sub-sub-supra condition in Experiment 2. (D) The sub-supra-supra condition in Experiment 2.
